# Mobile Imaging–Based Machine Learning for Dental Caries, Sealants, and Fluorosis: Protocol for a Cross-Sectional Model Development and Validation Study

**DOI:** 10.2196/91239

**Published:** 2026-03-30

**Authors:** Sang Mok Park, Semin Kwon, Shaun G Hong, Yuhyun Ji, Sreeram P Nagappa, Jung Woo Leem, Mei Lin, Eugenio D Beltrán-Aguilar, Susan O Griffin, Young L Kim

**Affiliations:** 1 Weldon School of Biomedical Engineering Purdue University West Lafayette, IN United States; 2 Dental Public Health Consultant Atlanta, GA United States; 3 Department of Epidemiology and Health Promotion New York University New York, NY United States; 4 Department of Oral Health Sciences, Kornberg School of Dentistry Temple University Philadelphia, PA United States; 5 TIS Consulting Group State College, PA United States; 6 Purdue Institute for Cancer Research Purdue University West Lafayette, IN United States; 7 Regenstrief Center for Healthcare Engineering Purdue University West Lafayette, IN United States

**Keywords:** mobile health, machine learning, computer vision, dental caries, dental fluorosis, dental sealants, intraoral camera, smartphone imaging, adolescent oral health

## Abstract

**Background:**

Assessing dental caries, sealants, and fluorosis is essential for public health surveillance, providing critical data to evaluate national prevention programs. Standard methods performed by dental professionals are often limited by affordability, accessibility, and scalability for both population-level and individualized assessments. Mobile health (mHealth) approaches to concurrently detect caries, sealants, and fluorosis have remained largely unexplored, especially at the population level.

**Objective:**

This study leverages mHealth technologies that integrate computer vision using machine learning and deep learning with images captured by smartphone cameras and low-cost intraoral cameras. The primary objective is to develop and validate models for detecting caries lesions, identifying sealants, and quantifying fluorosis severity from standardized dental images, using standardized visual clinical examinations as the reference standard.

**Methods:**

The proposed study population will include approximately 1000 adolescents in Colorado, United States, living in communities with naturally elevated fluoride levels in the public water system. Participants will undergo standardized clinical dental examinations and imaging using intraoral cameras and smartphones. Supervised learning models will incorporate reference chart–based color correction, radiomic spatial and textural features, and neural network classifiers. The reference standard will be standardized visual clinical examinations performed by trained and calibrated dental professionals. Two models will be developed and evaluated: one to detect caries lesions and sealants and another to assess fluorosis severity. Model performance will be evaluated against clinical assessments by dental professionals using stratified cross-validation and multiclass performance metrics while minimizing bias and accounting for confounders common to human examiners.

**Results:**

A standardized dental examination, an intraoral imaging protocol, and a smartphone imaging protocol are used to assess all 8 permanent molars for caries and sealants, as well as the 6 upper anterior teeth for fluorosis severity. Pilot studies were conducted to test study logistics and calibrate 3 examiners in person, supplemented by debriefings, mobile app training, and a web-based calibration module. The study was funded in September 2022 with supplemental funding awarded in June 2024. The study launched in May 2024, and as of January 2026, data have been collected from approximately 300 participants.

**Conclusions:**

The integration of computer vision and mobile device imaging will enable affordable, scalable, population-level assessments for detecting caries and sealants and quantifying fluorosis severity among adolescents. mHealth technologies have been increasingly incorporated into dentistry for both clinical decision support and at-home use. This protocol will further help establish a structured methodological framework for acquiring, processing, and analyzing mobile imaging data for dental health surveillance and epidemiological studies.

**International Registered Report Identifier (IRRID):**

DERR1-10.2196/91239

## Introduction

Dental caries (tooth decay) remains one of the most prevalent diseases of childhood [1,2]. If left untreated or unmanaged, it can impair a child’s ability to eat, speak, learn, and play [3-10]. Importantly, tooth decay is largely preventable with the use of sealants and fluorides. Reducing the burden of tooth decay and improving access to sealants and community water fluoridation are national health priorities [11-15]. However, excessive fluoride intake from drinking water, toothpaste, and other sources during the critical period of tooth development (up to approximately 6-8 years of age for most teeth) increases the risk of fluorosis, which manifests as enamel changes ranging from faint white striations in mild cases to pitting in severe forms [11-15].

Monitoring the prevalence of caries, sealants, and fluorosis has primarily been conducted at the national level through the National Health and Nutrition Examination Survey (NHANES) [16]. Some states have also collected data on caries and sealants using the Basic Screening Survey [17]. These surveillance efforts are costly, as they require trained dental professionals and portable examination equipment. Moreover, decisions regarding public health sealant programs and community water fluoridation are typically made at the local level, where relevant data are often limited or unavailable. As a result, affordable, valid, and reliable alternatives to traditional surveillance methods are urgently needed [18].

Dental clinical photography plays a critical role in diagnosis, monitoring, and treatment management [19-29]. Digital and mobile dental health applications using mobile devices (eg, smartphones and tablets) are increasingly used in telemedicine and remote care, particularly in home-based settings and in regions with limited access to dental care infrastructure [30-34]. Mobile health (mHealth) approaches leverage smartphones, tablets, and intraoral cameras to facilitate screening, treatment planning, remote examinations, and patient education, particularly in resource-limited settings and underserved populations [35,36]. Mobile devices are also well-suited for population-based dental public health surveillance in diverse settings at both local and national levels. The use of low-cost intraoral cameras and smartphones offers practical advantages for scalability, as these tools are widely available, user-friendly, and far less resource intensive than traditional mobile dental units and examinations performed by dental professionals. Their cost-effectiveness makes them especially attractive for public health programs operating under tight budget constraints.

Automated computer vision using machine learning and deep learning is an active area of research in oral health, often focused on caries detection [37-43]. However, evidence supporting the validity and feasibility of computer vision–integrated mHealth tools for jointly detecting caries, sealants, and fluorosis remains limited, especially for population-level use. Fluorosis detection, in particular, requires extensive examiner training and standardization, as current assessment measures are subjective [44,45]. Building on the promise of mHealth dental applications for both large-scale screening and individualized care [46-51], the mobility, simplicity, and affordability of mobile devices further support the integration of machine learning into population-level surveillance and public health monitoring. To date, no comprehensive mHealth protocol has proposed computer vision methods that combine caries and sealant detection using affordable intraoral cameras with smartphone fluorosis assessment for population-level surveillance and epidemiological studies.

The primary objective of this study is to develop and validate machine learning models using dental images for assessing dental health among middle- and high-school students (aged approximately 13-15 years), compared with the status quo of standardized visual clinical examinations. Specifically, the study aims to develop automated models for (1) detecting caries lesions and sealants and (2) detecting the presence and severity of fluorosis. With an emphasis on population-level surveillance, the models will focus on (1) caries and sealants in the permanent first and second molars and (2) fluorosis in the upper anterior teeth using Dean's Index–based fluorosis ratings [45,52].

The central hypothesis is that supervised learning models trained on intraoral camera images of molar occlusal surfaces and smartphone images of the upper anterior teeth can achieve detection performance comparable to the status quo of direct clinical evaluation by dental professionals. The reference standard for the supervised learning models will be the results of standardized visual clinical examinations performed by trained and calibrated dental professionals. Affordable intraoral cameras will be used for assessing posterior teeth due to limited physical space, as they provide higher diagnostic accuracy for caries than onboard smartphone cameras [53]. For fluorosis detection in anterior teeth, color correction using an application-specific color reference chart can enable reliable assessments with conventional smartphone cameras [54,55]. Smartphones are also chosen for their scalability and efficiency, as a single photograph can capture multiple anterior teeth simultaneously, supporting high-throughput population-level surveillance.

## Methods

### Overall Study Design

This is a noninterventional, cross-sectional observational study involving the acquisition of intraoral camera images of molar occlusal surfaces and smartphone images of the upper anterior teeth alongside standardized visual clinical examinations in adolescents. Assessments of caries and sealants and fluorosis severity will be used solely for research purposes. All data collection will take place during a single study visit; thus, a retention plan is not required.

### Setting and Recruitment

Data will be collected in selected middle and high schools from communities in Colorado, United States, with natural fluoride levels higher than 1 ppm to ensure a wide range of clinical presentations. According to data from the US Centers for Disease Control and Prevention (CDC) and the National Center for Education Statistics [56], 25 counties in Colorado meet these criteria, each with at least one qualifying water system and one school. Participant recruitment will be supported through coordination with school administrators and local community health coordinators via informational flyers, emails, and brief in-person presentations.

Caries and sealants are common among adolescents aged 12 to 15 years with a prevalence of 56.8% and 51.7%, respectively [57], whereas moderate to severe fluorosis affects fewer than 4% of US adolescents overall [58]. As fluorosis prevalence and severity increase with higher fluoride exposure, recruitment from areas with drinking water fluoride concentrations of ≥1.0 ppm is needed to obtain an adequate number of moderate or severe fluorosis images for model training. Among the 20 state oral health programs funded by the CDC, Colorado serves the largest population on public water systems with higher naturally occurring fluoride, and some of the earliest studies linking water fluoride content to fluorosis severity were conducted in these counties [52].

### Study Participants

The study aims to enroll up to 1000 students between the ages of 13 and 15 years. This age group is of particular interest due to the high prevalence of caries, the likelihood that sealants have already been applied, and the full eruption of the upper anterior teeth with varying degrees of fluorosis. In addition, adolescents in this age range are typically better able to follow instructions and cooperate during data collection procedures, enabling effective implementation of standardized protocols for both intraoral camera imaging and smartphone imaging.

The proposed sample size is anticipated to provide a sufficient number of outcome-positive cases to train the proposed models, mitigate overfitting risk, and support robust validation. We will split the dataset at the participant level (70% for training and 30% for testing); thus, a target sample size of 1000 yields approximately 700 participants for model development and approximately 300 independent participants for separate testing. The testing dataset will not be used or seen during model training and learning. This split helps ensure the test set contains enough positive cases while preventing data leakage during model selection. In similar photo-based studies, we successfully developed and validated machine learning models using data from approximately 400 to 500 participants [59,60].

### Inclusion and Exclusion Criteria

Consenting participants must agree to have digital images of their teeth captured using an intraoral camera and a smartphone. Potential participants will be excluded if they have fixed orthodontic appliances or other dental treatments that obstruct clear visualization of the anterior buccal and posterior occlusal tooth surfaces or if they have underlying medical conditions that make it unsafe or impractical to conduct dental assessments in a school setting (eg, conditions requiring antibiotic prophylaxis that cannot be administered onsite). These criteria ensure that all participants can safely and comfortably complete the clinical and imaging components of the study.

### Standardized Clinical Evaluation Procedure

**Figure 1:**
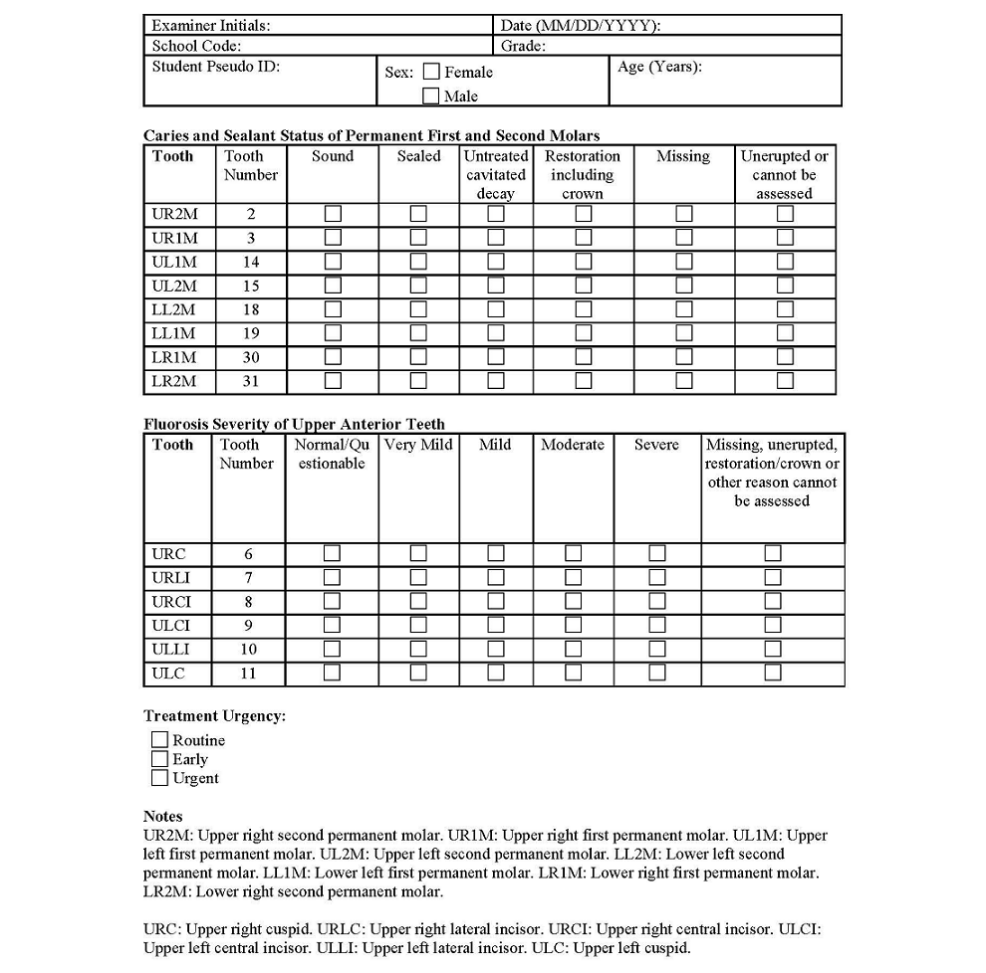
Dental screening form.

The clinical protocol is adapted from the dental examination method used in the NHANES, ensuring the standardized dental public health surveillance procedures and facilitating adequate access to the intraoral environment within a constrained time frame. First, participants will be given simple instructions on how to place a disposable dental retractor in the mouth to facilitate data collection. Second, the examiner will visually inspect all 8 permanent molars for caries lesions and the presence of sealants, categorizing each tooth as one of the following: sound; sealed; untreated cavitated decay; and restored, including crowns, missing, or unerupted or cannot be assessed. The occlusal surfaces of the molars will be dried using gauze prior to the assessments of caries and sealant presence. Third, the examiner will assess the 6 upper anterior teeth to determine fluorosis severity using the Dean's Index, classifying each tooth’s buccal surface as one of the following: normal/questionable; very mild; mild; moderate; severe; or cannot be assessed. Participants will be instructed to moisten the surfaces of the anterior teeth using their tongue and saliva in accordance with the Dean's Index evaluation protocol [45,52,61]. All clinical data will be registered in the data collection form (Figure 1).

### Intraoral Camera Imaging Procedure

**Figure 2:**
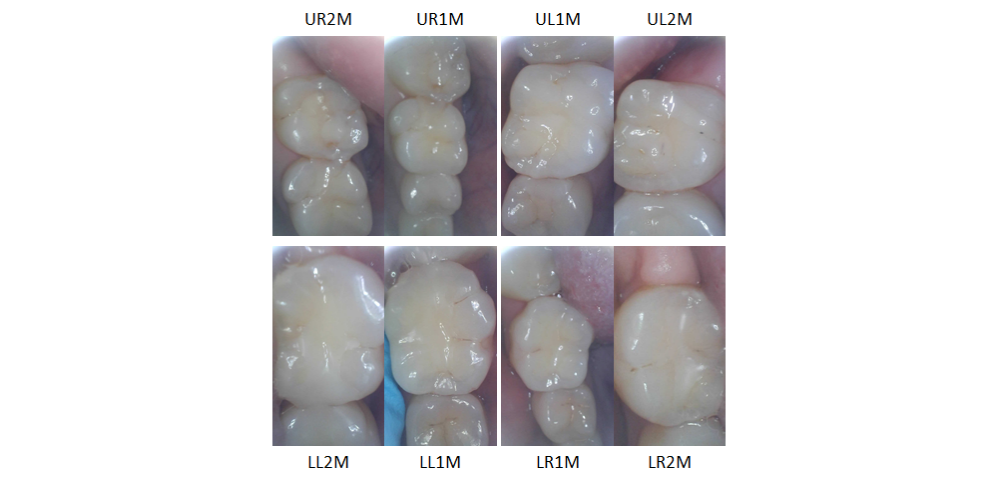
Representative images of the permanent molars. An affordable intraoral camera, wirelessly connected to a smartphone, captures detailed images of the occlusal surfaces of permanent molars. Computer vision and machine learning models using intraoral camera images are developed to automatically and interpretably detect caries and sealants. LL1M: lower left first molar; LL2M: lower left second molar; LR1M: lower right first molar; LR2M: lower right second molar; UL1M: upper left first molar; UL2M: upper left second molar; UR1M: upper right first molar; UR2M: upper right second molar.

The examiner will capture images of the occlusal surfaces of the molars using an intraoral camera wirelessly connected to a smartphone. For each participant, the examiner will capture 2 to 3 images of each of the 8 permanent molars after drying the teeth with gauze, generating a total of approximately 24 images per participant. A disposable, transparent protective sleeve will be fitted over the distal tip of the intraoral camera for infection control purposes. To ensure affordability and scalability, we selected a consumer-grade intraoral camera (Protector International) designed for home use. The device includes a built-in white LED light source and is available for approximately US $50 per unit. As a key specification, we evaluated the spatial resolution of an intraoral camera using the edge method [62,63], resulting in a spatial resolution of 37 μm. In a pilot study conducted as part of the data collection project described in Multimedia Appendix 1, images taken by an intraoral camera with the protective sleeve in place provided a clear visualization of the occlusal surfaces, which is essential for detecting caries and assessing sealants (Figure 2).

### Smartphone Camera Imaging Procedure

**Figure 3:**
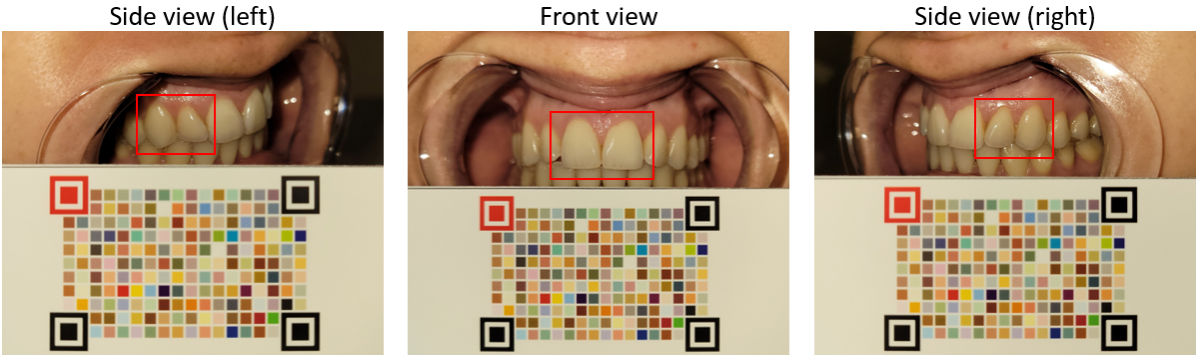
Representative images of upper anterior teeth alongside a customized color chart. A conventional smartphone camera captures detailed images of a participant’s anterior teeth with a retractor in place. The customized color chart is used to recover the absolute color of the teeth, regardless of device models, light conditions, or image file formats. Computer vision and machine learning models using smartphone camera images are developed to automatically and interpretably assess fluorosis severity.

The examiner will take images of the 6 upper anterior teeth from 3 angles (left, front, and right) using a smartphone, immediately after the participant moistens the surfaces of their teeth using their tongue and saliva, with the retractor in place. The front-angle image will capture the 2 central incisors, while the left and right angles will each include one lateral incisor and one cuspid (Figure 3). For each participant, 2 to 3 images will be taken from each of the 3 angles, resulting in a total of approximately 9 images per participant. We evaluated the spatial resolution of a typical smartphone camera (Samsung Galaxy S21) that will be used for the study, using the edge method in a laboratory setting [62,63], yielding a spatial resolution of 170 μm at a typical distance of 100 mm between the camera and the participant. Images captured using smartphone cameras can exhibit color variability due to differences in device models, light conditions, and image file formats. Achieving accurate and precise color quantification is a complex task that goes beyond simple white balancing or perceptual color constancy [54,64], requiring high-fidelity color standardization and canonicalization.

The image acquisition protocol will incorporate a custom-designed color reference chart to enable the recovery of absolute color values and the quantification of color changes for fluorosis assessment (Figure 3). Instead of commercially available color reference charts (eg, Macbeth ColorChecker or ColorChecker Classic Mini), a disposable custom-designed color chart will be used, which can be mass-produced using a commercially available inkjet printer [55]. As implemented in our previous studies [54,55], the customized color reference charts will be printed using the same inkjet printer (Canon imagePROGRAF PRO-1000) and the manufacturer-recommended paper (Canon Photo Paper Premium Fine Art Smooth). Print-to-print reproducibility and photostability testing of the printed charts support high consistency across printing batches and strong resistance to color fading under prolonged light exposure, demonstrating the chart’s practicality and scalability. Approximately the size of a business card, this chart is designed to be included in each image to facilitate color correction. To reduce the risk of infection and ensure accurate participant tracking, each color chart will include a participant ID number and will be appropriately disposed of after use. In a pilot study conducted as part of the data collection project described in Multimedia Appendix 1, images of the anterior teeth, juxtaposed with the customized color chart and taken with a Samsung Galaxy S21, provided a clear visualization of the anterior teeth from left, front, and right angles.

### Data Collection Mobile App

We will use a custom-developed data collection mobile app to facilitate the collection and secure transfer of dental images in a similar manner to our previous study [65]. The app enables examiners to upload both intraoral camera and smartphone images, which are transferred to a Health Insurance Portability and Accountability Act (HIPAA)–compliant cloud server and accessed through a high-security portal. The app requires completion of designated form fields before selecting photos from the smartphone gallery for upload. The app also supports offline functionality: in the event of network interruption, images are temporarily saved in a local folder and automatically uploaded in the background, one at a time, once connectivity is restored to minimize data congestion. Once all uploads are complete, the temporary folder is deleted from the device.

### Training and Calibration of Dental Examiners

**Figure 4:**
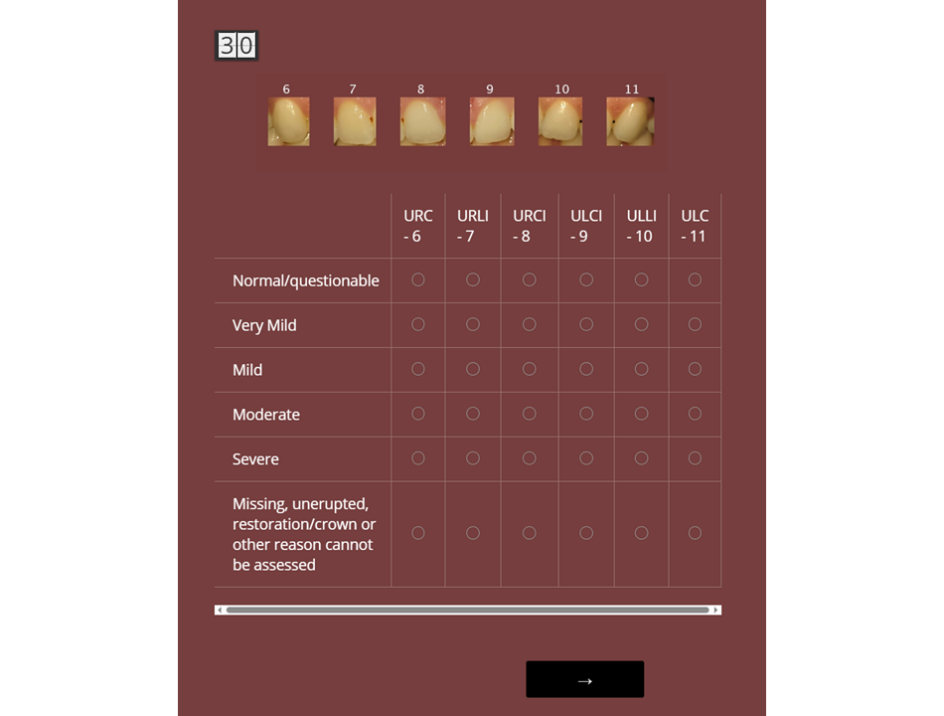
Representative web-based calibration module using Qualtrics. Field dental examiners evaluate color-corrected images on a home computer monitor under standardized viewing conditions. Evaluations are time-limited to mimic in-person participant assessments. This module uses a gingiva-like background color, and tooth images are color corrected using the custom color reference chart to ensure visual consistency. URC-6: upper right canine; URLI-7: upper right lateral incisor; URCI-8: upper right central incisor; ULCI-9: upper left central incisor; ULLI-10: upper left lateral incisor; ULC-11: upper left canine.

Field dental examiners will be trained using standardized procedures to ensure consistent application of assessment criteria for identifying the presence of caries lesions, sealants, and fluorosis, in accordance with the protocols used by the NHANES [66]. Before data collection, all field examiners will complete a 3-day training program led by a designated reference examiner. To support multiple sites and examiners, we will develop a web-based calibration module in Qualtrics (Qualtrics, LLC; Figure 4) in which examiners will evaluate dental images for caries and fluorosis on a monitor at home under controlled conditions (with recommended monitor display and ambient light settings). The purpose of this web-based calibration is to ensure that color-corrected image evaluations align with examiners’ in-person visual assessments and to enable examiners to calibrate against the reference examiner’s image-reading evaluations. The evaluation time will be limited to 30 seconds for all 6 teeth to mimic the timing of in-person assessments. A background color similar to gingival tissue will be used in the web-based interface, and the tooth images will be color corrected using the custom color reference chart to ensure color consistency.

### mHealth Detection Model Development

First, we will develop several mHealth detection models for caries and sealants using computer vision and machine learning approaches. The input will consist of intraoral camera images of the occlusal surfaces of the molars. The output of supervised learning models will be the clinical evaluation categorizing each tooth as one of the following: sound; sealed; untreated cavitated decay; or restored, including crowns, missing, or unerupted or cannot be assessed. We will leverage convolutional neural networks (CNNs), including the Visual Geometry Group network, a mask region–based CNN, and a residual network. We will apply data augmentation methods, including brightness, contrast, and saturation adjustments, as well as image flipping and random rotations. Our transfer learning strategy will incorporate pretrained models based on large-scale datasets for object detection, segmentation, and captioning, such as the Microsoft Common Objects in Context dataset and the ImageNet Large Scale Visual Recognition Challenge dataset. In addition, we will use radiomics, a technique widely applied across various medical imaging modalities. Radiomics enables the extraction of detailed spatial features (eg, texture, shape, and intensity) through predefined mathematical descriptors, providing insights beyond direct visual interpretation [59,60].

Second, we will develop a series of detection models for fluorosis using computer vision and machine learning approaches. The input will consist of smartphone images of the anterior teeth, and the output from supervised learning models will be fluorosis severity classifications based on the Dean's Index. Each tooth will be categorized as one of the following: normal/questionable; very mild; mild; moderate; severe; or cannot be assessed. The fluorosis assessment pipeline will include two main steps: (1) color correction will be applied to extract absolute color representations of the anterior teeth, ensuring consistency across different smartphone models, light conditions, and image file formats [54,55], and (2) CNN-based models will be trained using data augmentation and transfer learning to improve their robustness and accuracy. In addition, radiomics-based models will be developed to extract spatial and textural features specific to fluorosis assessment [59,60].

### Performance Evaluation

We will evaluate the models exclusively on the testing dataset (30%) to prevent data leakage and reduce the risk of overfitting. The testing dataset refers to an independent dataset reserved for external validation and will not be used or accessed during model training or development. Cross-validation will also be performed to evaluate model performance across different training data subsets. Specifically, k-fold cross-validation will be performed within the training dataset by partitioning the data into k approximately equal folds. In each iteration, the model will be trained on k−1 folds and evaluated on the remaining fold, rotating so that each fold serves as the validation set once. Performance will be summarized by averaging metrics across the k iterations, providing a robust estimate of model performance and supporting model selection and hyperparameter tuning. The held-out testing dataset will remain fully independent for final evaluation.

We will ensure comprehensive evaluations of each model’s performance across all classes in the multiclass classification tasks against the reference standard performed by trained and calibrated dental professionals [67,68]. While accuracy provides a basic measure of performance, representing the proportion of correctly predicted instances across all classes, it can be misleading in the presence of class imbalance. Thus, precision, recall, and *F*_1_-score will be computed for each class, along with their macro, micro, and weighted averages. Specifically, macroaveraging treats all classes equally, microaveraging aggregates contributions across all classes, and weighted averaging adjusts for class imbalance by weighting each class according to its support. In addition, confusion matrices will be used to visualize patterns of misclassification and to understand class-specific model performance better. Furthermore, TRIPOD-AI (Transparent Reporting of a Multivariable Prediction Model for Individual Prognosis or Diagnosis-Artificial Intelligence) [69] and CREMLS (Consolidated Reporting Guidelines for Prognostic and Diagnostic Machine Learning Modeling Studies: Development and Validation) [70] will be used for reporting our machine learning−based diagnostic results.

### Confidentiality, Data Storage, and Security

All study data will be stored and accessed in compliance with the HIPAA regulations. Specifically, image data will be labeled using participant identification numbers to maintain confidentiality. Demographic and clinical information recorded on paper forms by site personnel will be scanned and uploaded via the custom data collection app developed for this study, which transmits information to the secure HIPAA-compliant server. Access to the server is restricted to study investigators and authorized personnel. Electronic records will be stored on password-protected systems, and physical paper records will be kept in locked cabinets accessible only to authorized study staff.

### Ethical Considerations

This study has been approved by the CDC Institutional Review Board (protocol 7494). Purdue University and the Colorado Department of Public Health and Environment are under a deferral to the CDC Institutional Review Board. Our study involves recruiting participants from vulnerable populations aged 13 to 15 years. Written consent will be obtained from parents or legal guardians, and written assent will be required from all adolescent participants to confirm their willingness to participate. This approach ensures clear communication about the study’s objectives, procedures, and privacy protections, fostering a supportive and well-informed environment for both students and their families. All collected data will be deidentified and cannot be linked to individual participants. Participants will receive a free dental examination and an oral hygiene kit (including a toothbrush, toothpaste, and dental floss) as a token of appreciation for their participation.

## Results

### Protocol Development

We established the standardized school-based dental examination to assess all 8 permanent molars for caries and sealants and the 6 upper anterior teeth for fluorosis severity. The intraoral imaging protocol was developed in which examiners capture occlusal-surface images of the molars using an intraoral camera wirelessly connected to a smartphone (Figure 2). The smartphone imaging protocol was also established for capturing images of the 6 upper anterior teeth from 3 angles (ie, left, front, and right; Figure 3).

### Pilot Studies

We conducted pilot studies in Colorado communities in May and September 2024. The first pilot study focused on test runs of the study logistics and provided in-person, hands-on training in the procedures for 3 dental hygienists as field dental examiners. Using printed images representing diverse cases of fluorosis, caries, and sealants, as well as 15 volunteer participants, a certified and experienced dental clinical and surveillance expert provided examiner training and calibration. The second pilot study further ensured examiner training and calibration in school-based settings over 3 days. It involved 55 volunteer participants and focused on acquiring high-quality photos and practicing data upload to the designated server. Debriefing sessions helped ensure that the clinical examiners were consistent and calibrated. During the pilot studies, the examiners also received training on how to use the app for data transfer. In addition, using this web-based calibration module (Figure 4), we verified that the expert’s color-corrected image evaluations aligned with the expert’s in-person visual assessments. We also validated that dental field examiners could score dental caries, sealant presence, and fluorosis severity across a range of cases using color-corrected images with expert feedback provided to refine scoring consistency.

### Status

The procedure and protocol development were initially funded in September 2022, and supplemental funding was awarded in June 2024. The data collection began in May 2024. As of January 2026, approximately 300 participants had been enrolled. Results will be disseminated following completion of target enrollment and model development.

## Discussion

To the best of our knowledge, this will be the first study to develop computer vision models for the joint surveillance of dental caries, sealants, and fluorosis severity using mobile devices among adolescents. Specifically, computer vision–assisted surveillance of fluorosis will be critical for monitoring population-level impact. The US Public Health Service (USPHS) advises that US Department of Health and Human Services agencies continue to prioritize the development of valid and reliable measures for fluorosis assessment. In December 2012, the USPHS revised its recommendation for the optimal fluoride concentration in drinking water to 0.7 ppm for the prevention of tooth decay [11]. This level was intended to balance protection against caries with minimizing the risk of fluorosis, emphasizing the importance of monitoring both caries and fluorosis. In population-level studies, fluorosis assessment is typically performed by human examiners; however, examiner training and standardization are complex and often introduce bias, even when image-based scoring methods are used [45,71]. Recent cycles of US fluorosis data have exhibited substantial variation across survey cycles that cannot be explained by changes in national fluoride intake [16,18,58,72], indicating a need for more objective assessments.

Most previous studies using mobile devices, with or without computer vision (machine learning or deep learning), have focused on caries detection in resource-limited settings, including at-home and rural environments. Multiple studies have validated the use of smartphone—and intraoral camera—acquired images for assessing caries in both children and older adults with interpretation by dental professionals [37-41,73-77]. The use of machine learning for reliable caries detection has also been demonstrated in at-home settings [36,39,40,78-83]. For example, a study of parent-child dyads has demonstrated the feasibility of using smartphone images of the anterior and posterior teeth for at-home detection of caries with high parental acceptability [41,84]. Another study using intraoral images has shown that a deep learning model can simultaneously detect caries and fissure sealants with performance comparable to early-career dentists [42]. In addition, fluorescence imaging and other optical modalities have been explored for fluorosis detection [85,86], but these methods have yet to be applied in real-world field settings. Upon successful completion of this study, mHealth technologies can potentially be integrated into existing national surveillance efforts to strengthen the monitoring of progress toward national oral health objectives. Combining computer vision with clinical photos from mobile devices can further support low-cost, scalable, population-wide screening to detect caries, identify sealant presence, and assess fluorosis severity.

## Appendix

Multimedia Appendix 1 Federal Register notice describing the broader data collection project underlying this study: evaluating deep learning algorithm assessment of digital photographs for dental public health surveillance. Department of Health and Human Services, Centers for Disease Control and Prevention.

## Abbreviations

CDC: Centers for Disease Control and Prevention
CNN: convolutional neural network
CREMLS: Consolidated Reporting Guidelines for Prognostic and Diagnostic Machine Learning Modeling Studies: Development and Validation
HIPAA: Health Insurance Portability and Accountability Act
mHealth: mobile health
NHANES: National Health and Nutrition Examination Survey
TRIPOD-AI: Transparent Reporting of a Multivariable Prediction Model for Individual Prognosis or Diagnosis-Artificial Intelligence
USPHS: US Public Health Service

## Data Availability

Data sharing is not applicable to this paper, as no datasets were analyzed during this study.
