# Supplementary material for: Mobile Imaging–Based Machine Learning for Dental Caries, Sealants, and Fluorosis: Protocol for a Cross-Sectional Model Development and Validation Study
Source: JMIR Res Protoc. 2026 Mar 30;15:e91239. doi: 10.2196/91239 (PMC13077280; doi:10.2196/91239)
Supplement: Multimedia Appendix 1 [file resprot_v15i1e91239_app1.pdf]

AUTHENTICATED U.S. GOVERNMENT INFORMATION GPO

7. May not use Federal funds from a contract to develop Challenge applications or to fund efforts in support of a Challenge submission.

8. Shall not be deemed ineligible because the individual or entity used Federal facilities or consulted with Federal employees during a competition if the facilities and employees are made equitably available to all individuals and entities participating in the competition.

9. Shall not be required to purchase liability insurance as a condition of participation in this competition.

Additional Rules of Participation

By participating in this Challenge, each individual (whether participating singly or in a group) or entity:

1. Agrees to follow all applicable federal, state, and local laws, regulations, and policies.

2. Agrees to comply with all terms and conditions of participation in this Challenge.

3. Agrees that the submission will not use HHS or AHRQ logos or official seals and will not claim endorsement by HHS or AHRQ.

4. Understands that all materials submitted to AHRQ as part of a submission become AHRQ records.

5. Agrees that the submission must not infringe upon copyright or any other rights of any third party.

6. Agrees to assume any and all risks and waive claims against the Federal Government and its related entities, except in the case of willful misconduct, for any injury, death, damage, or loss of property, revenue, or profits, whether direct, indirect, or consequential, arising from participation in this prize contest, whether the injury, death, damage, or loss arises through negligence or otherwise.

7. Agrees to indemnify the Federal Government against third-party claims for damages arising from or related to Challenge activities.

8. Understands that AHRQ reserves the right to cancel, suspend, and/or modify this prize contest, or any part of it, for any reason, at AHRQ's sole discretion. AHRQ also reserves the right not to award any prizes if no entries are deemed worthy.

9. Understands that AHRQ will not select a winner that is named on the Excluded Parties List System (EPLS).

Intellectual Property (IP) Rights

1. Each participant retains title and full ownership in and to their submission. Participants expressly reserve all intellectual property rights not expressly granted.

2. By participating in the Challenge, each participant (whether participating singly or in a group) acknowledges that he or she is the sole author or owner of, or has a right to use, any copyrightable works that the submission comprises, that the works are wholly original with the participant (or is an improved version of an existing work that the participant has sufficient rights to use and improve), and that the submission does not infringe any copyright or any other rights of any third party of which participant is aware. In addition, each participant (whether participating singly or in a group) grants to the U.S. Government a paid-up, nonexclusive, royalty-free, irrevocable worldwide license and the right to reproduce, publish, post, link to, share, display publicly (on the web or elsewhere) and prepare derivative works, including the right to authorize others to do so on behalf of the U.S. Government.

3. If the submission includes any third party works (such as third party content or open source code), the participant must be able to provide, upon request, documentation of all appropriate licenses and releases for use of such third-party works. If the participant cannot provide documentation of all required licenses and releases, AHRQ reserves the right, in its sole discretion, to disqualify the submission.

Dated: May 31, 2023.

Marquita Cullom,

Associate Director.

[FR Doc. 2023–11869 Filed 6–2–23; 8:45 am]

BILLING CODE 4160–90–P

DEPARTMENT OF HEALTH AND HUMAN SERVICES

Centers for Disease Control and Prevention

[60Day–23–23FJ; Docket No. CDC–2023–0042]

Proposed Data Collection Submitted for Public Comment and Recommendations

AGENCY: Centers for Disease Control and Prevention (CDC), Department of Health and Human Services (HHS).

ACTION: Notice with comment period.

SUMMARY: The Centers for Disease Control and Prevention (CDC), as part of its continuing effort to reduce public burden and maximize the utility of government information, invites the general public and other federal agencies the opportunity to comment on a proposed information collection, as required by the Paperwork Reduction Act of 1995. This notice invites comment on a proposed information collection project titled Evaluating Deep Learning Algorithm Assessment of Digital Photographs for Dental Public Health Surveillance. This project entails one-time data collection of oral health data from 1,000 school students to examine the feasibility and validity of using digital photos taken by non-dental professionals, which are analyzed by deep learning algorithms to assess youth's oral health status.

DATES: CDC must receive written comments on or before August 4, 2023.

ADDRESSES: You may submit comments, identified by Docket No. CDC–2023–0042 by any of the following methods:

- *Federal eRulemaking Portal: www.regulations.gov.* Follow the instructions for submitting comments.
- *Mail:* Jeffrey M. Zirger, Information Collection Review Office, Centers for Disease Control and Prevention, 1600 Clifton Road NE, MS H21–8, Atlanta, Georgia 30329.

Instructions: All submissions received must include the agency name and Docket Number. CDC will post, without change, all relevant comments to *www.regulations.gov.*

Please note: Submit all comments through the Federal eRulemaking portal (www.regulations.gov) or by U.S. mail to the address listed above.

FOR FURTHER INFORMATION CONTACT: To request more information on the proposed project or to obtain a copy of the information collection plan and instruments, contact Jeffrey M. Zirger, Information Collection Review Office, Centers for Disease Control and Prevention, 1600 Clifton Road NE, MS H21–8, Atlanta, Georgia 30329; Telephone: 404–639–7118; Email: *omb@cdc.gov.*

SUPPLEMENTARY INFORMATION: Under the Paperwork Reduction Act of 1995 (PRA) (44 U.S.C. 3501–3520), federal agencies must obtain approval from the Office of Management and Budget (OMB) for each collection of information they conduct or sponsor. In addition, the PRA also requires federal agencies to provide a 60-day notice in the **Federal Register** concerning each proposed collection of information, including each new proposed collection, each proposed extension of existing collection of information, and each reinstatement of previously approved information collection before submitting the collection to the OMB for approval. To comply with this requirement, we are publishing this notice of a proposed data collection as described below.

The OMB is particularly interested in comments that will help:

1. Evaluate whether the proposed collection of information is necessary

for the proper performance of the functions of the agency, including whether the information will have practical utility;

2. Evaluate the accuracy of the agency's estimate of the burden of the proposed collection of information, including the validity of the methodology and assumptions used;

3. Enhance the quality, utility, and clarity of the information to be collected;

4. Minimize the burden of the collection of information on those who are to respond, including through the use of appropriate automated, electronic, mechanical, or other technological collection techniques or other forms of information technology, *e.g.,* permitting electronic submissions of responses; and

5. Assess information collection costs.

Proposed Project

Evaluating Deep Learning Algorithm Assessment of Digital Photographs for Dental Public Health Surveillance—New—National Center for Chronic Disease Prevention and Health Promotion (NCCDPHP), Centers for Disease Control and Prevention (CDC).

Background and Brief Description

By age 19, 57% of U.S. adolescents have experienced tooth decay and 17% have at least one decayed tooth needing treatment. Prevalence of untreated tooth decay among non-Hispanic Black and Mexican American adolescents is about 30% higher than among non-Hispanic White adolescents, and among low-income, almost twice the prevalence of higher-income adolescents. Untreated tooth decay will not resolve and can cause pain, infection, and difficulties in learning. Poor oral health in youth is associated with both lower school attendance and grades. More than 34 million school hours are lost annually due to unplanned dental visits for acute care needs. Reducing the percentage of youths who have experienced tooth decay and the percentage with untreated tooth decay are national health goals (Healthy People 2030).

There are two highly effective interventions to prevent tooth decay. Dental sealants prevent about 80% of cavities over two years in the permanent molars where about 90% of tooth decay occurs. Fluoride can prevent decay in permanent teeth by 15% to 43% per year depending on mode of delivery. Although the American Dental Association recommends dentists provide topical fluoride and dental sealants to youth at risk for caries, uptake of these services is low with about 20% of low-income youth receiving them during an annual dental visit. Access to these preventive services as measured by dental sealant prevalence and receipt of preventive dental services among low-income children are national health goals.

The Centers for Disease Control and Prevention (CDC) has collected national data on caries, sealant, and fluorosis prevalence in the National Health and Nutrition Examination Survey (NHANES) for over 30 years and has supported state oral health programs to collect data on caries and sealant prevalence through cooperative agreements since 2001. Twenty states are currently funded from September 2018 to August 2023 by *Actions to Improve Oral Health Outcomes,* CDC–RFA–DP18–1810. Collecting these data can be resource intensive as they are obtained through visual/tactile examinations conducted by dental professionals. These data, however, have enabled federal and state agencies to: (1) prioritize groups at elevated risk for enhanced prevention efforts; (2) monitor trends in children's oral health status and disparities; (3) inform planning, implementation and evaluation of effective oral health interventions, programs, and policies; (4) measure progress toward Healthy People objectives; and (5) educate the public and policy makers regarding cross-cutting public health programs. Having local estimates of these measures would enable decision-makers to better prioritize communities for programs that increase access to preventive dental services.

CDC is examining the feasibility and validity of using digital photos taken by non-dental professionals, which in turn would be analyzed by deep learning algorithms to assess youth's oral health status in lieu of human examination. This deep learning assessment tool ultimately could be used by public health officials for dental public health surveillance at the local, state, and national level. It is anticipated that obtaining information on dental conditions via deep learning assessment of digital images as opposed to human assessment will: (1) be more cost-effective as it would not require dental personnel; and (2) improve the accuracy of assessment due to minimal bias and less confounding factors associated with the examiner (*e.g.,* subjective index and thresholding). This tool also would offer mobility, simplicity, and affordability for rapid and scalable adaptation in community-based settings.

In order to train and test the deep learning algorithms to identify caries, sealants, and fluorosis, data on these conditions as assessed by standardized examiners and corresponding photos are required. The CDC requests a one-year OMB approval for the one-time collection of oral health data from 1,000 middle- and high-school students in Colorado communities with naturally occurring fluoride in the tap water at or exceeding one part per million. The Colorado State Health Department will implement the collection by recruiting selected schools and dental examiners, gaining consent, arranging logistics, and collecting data from dental examination and photos taken by the dental examiners. CDC will provide dental examination and photo taking protocols and train the examiners. Data collected for each student will include: (1) human assessment of fluorosis severity in the six upper anterior teeth, and caries/sealant assessment of the occlusal surfaces of the eight permanent molars; and (2) nine smartphone digital photos of the upper anterior teeth and 24 intraoral camera digital photos of the occlusal surfaces of the eight permanent molars. Only de-identified data will be collected. All de-identified data—digital photos of the teeth and the completed paper screening form—will be uploaded to a HIPAA compliant cloud storage box that can only be accessed by examiners and designated CDC researchers with administrative rights. CDC is authorized to collect this information under the Public Health Service Act, title 42, section 247b–14, Oral health promotion and disease prevention; and the Public Health Service Act, title 42, section 301.

CDC proposes using data collected from 750 students to train the deep learning algorithms to assess caries, sealants, and fluorosis and data from 250 students to evaluate the accuracy of the algorithms in terms of agreement with standardized examiner assessment. Manuscripts on: (1) the methodologies used to ensure sufficient photo quality when taken under field conditions; and (2) the performance of the deep learning algorithms will be submitted to peer-reviewed journals. The deep learning tool if sufficiently accurate will be piloted in one data collection cycle of NHANES that is administered by the National Centers for Health Statistics (NCHS). Ultimately, the tool would be shared with the state and local oral health programs, the Association of State and Territorial Dental Directors, and other pertinent partners.

The CDC requests OMB clearance for data collection for one year. The total estimated annualized burden hours are 827. There are no costs to student respondents other than their time.

ESTIMATED ANNUALIZED BURDEN HOURS

Type of respondent	Form name	Number of respondents	Number of responses per respondent	Average burden per response (in hr)	Total burden (in hr)
Child	Screening/photo/form	1,000	1	16/60	270
Parent or caretaker	Consent	1,000	1	1/60	17
Screener	Screening/photo form includes training, travel, screening and photos, and ongoing technical assistance.	6	1	90	540
Total					827

Jeffrey M. Zirger,

Lead, Information Collection Review Office, Office of Public Health Ethics and Regulations, Office of Science, Centers for Disease Control and Prevention.

[FR Doc. 2023–11859 Filed 6–2–23; 8:45 am]

BILLING CODE 4163–18–P

DEPARTMENT OF HEALTH AND HUMAN SERVICES

Centers for Disease Control and Prevention

[60Day–23–1289; Docket No. CDC–2023–0041]

Proposed Data Collection Submitted for Public Comment and Recommendations

AGENCY: Centers for Disease Control and Prevention (CDC), Department of Health and Human Services (HHS).

ACTION: Notice with comment period.

SUMMARY: The Centers for Disease Control and Prevention (CDC), as part of its continuing effort to reduce public burden and maximize the utility of government information, invites the general public and other federal agencies the opportunity to comment on a continuing information collection, as required by the Paperwork Reduction Act of 1995. This notice invites comment on a proposed information collection project titled Sealant Efficiency Assessment for Locals and States (SEALS). This data will be collected from local school sealant programs to generate efficiency performance measures, which will allow CDC to identify feasible benchmarks and best practices contributing to school sealant program efficiency.

DATES: CDC must receive written comments on or before August 4, 2023.

ADDRESSES: You may submit comments, identified by Docket No. CDC–2023–0041 by any of the following methods:

- *Federal eRulemaking Portal: www.regulations.gov.* Follow the instructions for submitting comments.
- *Mail:* Jeffrey M. Zirger, Information Collection Review Office, Centers for Disease Control and Prevention, 1600 Clifton Road NE, MS H21–8, Atlanta, Georgia 30329.

Instructions: All submissions received must include the agency name and Docket Number. CDC will post, without change, all relevant comments to *www.regulations.gov.*

Please note: Submit all comments through the Federal eRulemaking portal (*www.regulations.gov*) or by U.S. mail to the address listed above.

FOR FURTHER INFORMATION CONTACT: To request more information on the proposed project or to obtain a copy of the information collection plan and instruments, contact Jeffrey M. Zirger, Information Collection Review Office, Centers for Disease Control and Prevention, 1600 Clifton Road NE, MS H21–8, Atlanta, Georgia 30329; Telephone: 404–639–7570; Email: *omb@cdc.gov.*

SUPPLEMENTARY INFORMATION: Under the Paperwork Reduction Act of 1995 (PRA) (44 U.S.C. 3501–3520), federal agencies must obtain approval from the Office of Management and Budget (OMB) for each collection of information they conduct or sponsor. In addition, the PRA also requires federal agencies to provide a 60-day notice in the **Federal Register** concerning each proposed collection of information, including each new proposed collection, each proposed extension of existing collection of information, and each reinstatement of previously approved information collection before submitting the collection to the OMB for approval. To comply with this requirement, we are publishing this notice of a proposed data collection as described below.

The OMB is particularly interested in comments that will help:

1. Evaluate whether the proposed collection of information is necessary for the proper performance of the functions of the agency, including whether the information will have practical utility;
2. Evaluate the accuracy of the agency's estimate of the burden of the proposed collection of information, including the validity of the methodology and assumptions used;
3. Enhance the quality, utility, and clarity of the information to be collected;
4. Minimize the burden of the collection of information on those who are to respond, including through the use of appropriate automated, electronic, mechanical, or other technological collection techniques or other forms of information technology, *e.g.,* permitting electronic submissions of responses; and
5. Assess information collection costs.

Proposed Project

Sealant Efficiency Assessment for Locals and States (SEALS) (OMB Control No. 0920–1289)—Reinstatement with change—National Center for Chronic Disease Prevention and Health Promotion (NCCDPHP), Centers for Disease Control and Prevention (CDC).

Background and Brief Description

By age 19, 67% of U.S. adolescents living in poverty have experienced tooth decay and 27% have at least one decayed tooth needing treatment. School sealant programs provide dental sealants, which protect against 80% of cavities for two years, and continue to protect against 50% of cavities for up to four years. CDC requests information from states regarding children's cavity risk, one-year sealant retention rate, sealant program services delivered, and school sealant program cost and quantity of resources used at each school event. This data will allow CDC and states to monitor the performance and efficiency of their school sealant programs, which will improve and extend program delivery to more children.

CDC requests OMB approval for a Reinstatement of a previously approved data collection. The total estimated
